# Challenging Cognitive Control by Mirrored Stimuli in Working Memory Matching

**DOI:** 10.3389/fpsyg.2017.00653

**Published:** 2017-04-28

**Authors:** Maria Wirth, Robert Gaschler

**Affiliations:** ^1^Department of Psychology, Universität LeipzigLeipzig, Germany; ^2^Department of Psychology, FernUniversität in HagenHagen, Germany; ^3^Interdisciplinary Research Cluster Image, Knowledge, Gestaltung, Humboldt-Universität BerlinBerlin, Germany

**Keywords:** mirror generalization, object recognition, cognitive conflict, cognitive control

## Abstract

Cognitive conflict has often been investigated by placing automatic processing originating from learned associations in competition with instructed task demands. Here we explore whether mirror generalization as a congenital mechanism can be employed to create cognitive conflict. Past research suggests that the visual system automatically generates an invariant representation of visual objects and their mirrored counterparts (i.e., mirror generalization), and especially so for lateral reversals (e.g., a cup seen from the left side vs. right side). Prior work suggests that mirror generalization can be reduced or even overcome by learning (i.e., for those visual objects for which it is not appropriate, such as letters d and b). We, therefore, minimized prior practice on resolving conflicts involving mirror generalization by using kanji stimuli as non-verbal and unfamiliar material. In a 1-back task, participants had to check a stream of kanji stimuli for identical repetitions and avoid miss-categorizing mirror reversed stimuli as exact repetitions. Consistent with previous work, lateral reversals led to profound slowing of reaction times and lower accuracy in Experiment 1. Yet, different from previous reports suggesting that lateral reversals lead to stronger conflict, similar slowing for vertical and horizontal mirror transformations was observed in Experiment 2. Taken together, the results suggest that transformations of visual stimuli can be employed to challenge cognitive control in the 1-back task.

## Introduction

Everyday life contains situations where mirror-reversed images need to be processed with care. When trying to identify one’s cup on a shelf, it should not matter whether the handle points left or right. Yet, categorizing the direction the handle is pointing, involves activation of spatial representations and can lead to processing costs (cf. Koch, 2009). In the current work we explore whether mirror generalization can be employed to challenge cognitive control in the lab.

Cognitive control is an umbrella term comprising a wide range of cognitive processes and behavioral competencies (Miyake et al., 2000; Chan et al., 2008). Cognitive control is needed in situations in which routine behavior is not sufficient to produce an adequate performance result or if conflicts in information processing are detected (e.g., Egner, 2008). Cognitive conflict is typically studied using stimuli that have different dimensions (or features), which are either relevant or irrelevant with respect to task demands (e.g., Ridderinkhof et al., 2004; Egner, 2008). Cognitive conflict has been shown to arise if different dimensions of stimuli or responses have a high degree of perceptual, conceptual, or structural similarity and also indicate competing response tendencies (e.g., Egner, 2007). The level of conflict depends on the extent to which deliberate and automatic stimulus processing and/or response selection are competing.

Inducing cognitive conflict by overlapping stimulus dimensions might have the disadvantage that it can be difficult to disentangle interference at the level of task relevant and irrelevant stimulus information from interference at the level of responses (e.g., Egner, 2008). In addition, most cognitive conflict tasks pit task demands against task inappropriate response tendencies acquired in the experiment (e.g., the combined method to assess volitional control proposed by Ach, 1910/2006; or the letter variant of the Eriksen Flanker Task, Eriksen and Eriksen, 1974; see also MacLeod and Dunbar, 1988), or learned before entering the experiment (e.g., color-naming in the Stroop Task; Stroop, 1935). Instead of relying on learned response tendencies, we explored whether mirror generalization as a congenital mechanism can be employed to create cognitive conflict. Based on the assumption that the visual system automatically generates an invariant representation of visual objects and their mirrored counterparts, we investigated if cognitive conflict arises if mirrored and non-mirrored stimuli require different responses despite that automatic processing would suggest the same response for the original and mirrored counterpart. Before we present our predictions, we will provide a review of past relevant work.

### Cognitive Conflict

Cognitive conflict can arise at different levels of information processing such as perceptual processing, stimulus categorization, or response selection (e.g., Botvinick et al., 2004). Most often, cognitive conflict is studied using speeded response tasks in which different, incompatible response channels are co-activated by conflicting information present in a stimulus (e.g., Carter and van Veen, 2007). If different stimulus dimensions are mapped onto the same response, responses are fast and accurate. If stimulus dimensions are mapped onto a different response, responses are slower and less accurate. For example, in the Eriksen Flanker Task, increased reaction times and error rates are observed if the centrally presented target stimulus is flanked by incongruent distractors (e.g., Eriksen and Eriksen, 1974; Eriksen and Schultz, 1979). In the Stroop Task (Stroop, 1935; MacLeod, 1991), font color naming latency is slowed when the font color is different from word content (e.g., the word “green” printed in red ink). In both tasks, conflict is supposed to be stimulus-based as it arises from stimulus-stimulus (S-S) overlap (Egner, 2008). In the Eriksen Flanker Task, task- relevant and task-irrelevant stimulus dimensions are drawn from the same set of symbols and in the Stroop Task, to-be-named ink color can be in conflict with the word meaning. It has been argued that interference tasks, such as the Stroop Task or the Eriksen Flanker Task, involve conflict not only at the stimulus level, but also at the response level (Kornblum et al., 1990, 1999; Kornblum, 1994; Zhang et al., 1999). This in turn entails that different mechanisms could be engaged in order to solve the cognitive conflict. Conflict between stimulus dimensions could be solved either by inhibiting irrelevant information or by excitatory biasing of the processing of task relevant stimulus features (e.g., Botvinick et al., 2001; Nieuwenhuis and Yeung, 2005). In addition, as proposed by different dual process models of stimulus-response (S-R) relations (e.g., Kornblum et al., 1990; De Jong et al., 1994; Eimer et al., 1995; Ridderinkhof et al., 1995), an active response inhibition mechanism could selectively reduce the activation of the incongruent response.

There are only few tasks that induce primarily response-based conflicts such as the go/no-go task (e.g., Donders, 1868/1969). The go/no-go task and other related response-based conflict tasks are based on establishing prepotent responses by, for example, presenting frequent S–R mappings. Performance is than dominated by these prepotent response tendencies to frequent stimuli, at a cost of decreased performance in infrequent trials (e.g., Carter and van Veen, 2007). During no-go trials, the prepotent go response has to compete with the tendency to withhold responding, resulting in false alarms in no-go trials (Jones et al., 2002). A problem of these tasks is that they rely on the idea that through instruction and practice comparable response tendencies are established in different subjects. However, subjects differ in the extent of their experiences and learning abilities which they bring to the task (cf. Krüger and Suchan, 2016) and, therefore, can differ widely in their learning and forgetting curves (e.g., Nembhard and Uzumeri, 2000; Brown and Heathcote, 2003) that might modulate task processing. The amount of experimental control to be gained by employing automatic processing tendencies acquired in the lab or acquired before entering the lab might, thus, be limited. This suggests to explore whether congenital mechanisms (c.f. Pegado et al., 2011) can be employed to challenge cognitive control instead.

To summarize, in frequently used conflict paradigms the distinction between stimulus and response conflict is rather difficult. The amount of cognitive conflict in these tasks may also be influenced by interindividual differences in task set acquisition.

### Mirror Generalization

Mirror generalization allows humans to recognize objects (e.g., animals, tools, or faces) irrespective of their orientation. Mirror generalization is thought to be an adaptive mode of processing visual information (e.g., Bornstein et al., 1978), especially in situations of threat. For example, a tiger needs to be recognized as a tiger, regardless of it facing left or right (e.g., Rollenhagen and Olson, 2000). Mirror generalization can be helpful in everyday activities such as recognizing a tool regardless of its handle pointing left or right. However, as objects and mirror images of these objects are encoded in the same way in the ventral visual stream (Dehaene et al., 2010), mirror invariance can be detrimental for the recognition of objects such as letters and words that have distinct alignments (e.g., Caramazza and Hillis, 1990). Especially the tolerance to lateral reversals such as b/d or p/q can cause severe problems as enantiomorphs will be identified as the same letter (Dehaene et al., 2010). Mirror errors such as confusion between b/d are not exceptional in young primary school children (e.g., Schott, 2007). In addition, not just letters and words are mirrored but also digits such as ƹ/3 (e.g., Fischer, 2011). Although it is assumed that through schooling, the mirror invariance characteristics of the visual system are unlearned or inhibited for written language (e.g., Lachmann and van Leeuwen, 2007; Dehaene et al., 2010; Duñabeitia et al., 2011; Kolinsky et al., 2011), recent evidence suggests that expert readers never completely “unlearn” the mirror-generalization process (Borst et al., 2015). In addition, previous research has shown that mirror generalization has a profound influence on the processing of pictures (faces, tools, and animals; e.g., Duñabeitia et al., 2011) as well as words (e.g., Poldrack et al., 1998). The visual recognition of word or letter enantiomorphs results in a slowing of the identification process and a loss of accuracy (e.g., Poldrack et al., 1998; Perea et al., 2011). The slowed responses for the differentiation of enantiomorphs could arise from conflict between the tendency of the brain to treat mirror-images as equivalent and the task requirements to differentiate them. If a task choice is based upon the identity of similar but mirrored images, two mutually exclusive response tendencies should arise – one generated from the more automatic mirror generalization process and one originating from more deliberate object identification processes.

Most studies that investigated the effect of mirror generalization on the discrimination of visual objects have focused on the processing of letters or words or have presented a small number of objects such as animals (e.g., Ryan and Schnyer, 2007; Dehaene et al., 2010; Duñabeitia et al., 2011; Perea et al., 2011; Borst et al., 2015). For example, in a negative priming experiment, Borst et al. (2015) presented letters with mirror image counterparts (b/d) and showed that this affected the discrimination of animals that were mirror images of each other. However, the interpretation of the previous results may be limited given the following problems related to familiarity with the presented stimuli: (1) Mirror generalization is not very effective if only a small number of stimuli is presented (e.g., Boldt et al., 2013). (2) Alphabetic words may have phonetic and semantic influences on stimuli processing. (3) Mirrored letters in words also include inconsistencies in reading directions which should make it easier to spot them. Given these limitations of previous studies, it is an open question if mirror generalization is suitable for inducing cognitive conflict.

Taken together, the innate property of the visual system to treat mirror reversed objects as identical is detrimental if mirror pairs have to be distinguished. There has been no systematic investigation if mirror generalization is suitable for inducing cognitive conflict.

### The Present Study

The main aim of the study was to implement a task in which a cognitive conflict was induced by the demand to discriminate mirrored and non-mirrored stimuli. Given the mixture of stimulus- and response-based conflicts in many choice reaction time tasks, we excluded interferences from any direct stimulus-based competition by presenting one stimulus at a time. We accomplished this by creating a *N*-back task, in which participants were presented with a stream of visual stimuli (Kirchner, 1958). In this task, participants indicated when the current stimulus matched the one presented in the previous trial. In most *N*-back tasks, participants are asked to indicate if the current stimulus is an exact repetition of the previous stimulus (*target*) or not (*different*). Our task also included comparisons between lateral reversals of the same stimulus (*vertical*). Because previous studies have shown that errors in mirror image discrimination are mainly driven by vertical enantiomorphs (e.g., Kolinsky et al., 2011), up-down mirror images of each other (*horizontal*) were included to test the uniqueness of vertical mirror generalization effects. In order to overcome limitations of previous studies such as high familiarity of linguistic stimuli or small item sets, Japanese characters (kanji) were used as stimuli. They are complex graphic forms for participants naïve to the language and are neither associated with semantic nor phonetic representations (Ueki et al., 2006). Kanji are not fully symmetrical, which allows to mirror them without obtaining identical images. In addition participants are unfamiliar with kanji. Given these characteristics, kanji are suitable stimuli for our experiments because mirroring these stimuli produces images that cannot be distinguished easily from the original.

In two experiments, participants had to discriminate between exact repetitions and alternations in the 1-back task. If a trial showed the lateral reversal of the n-1 image, the participant was to classify this as an alternation. Automatic mirror generalization should complicate complying to this instruction. This prediction was supported by previous evidence indicating that mirror image invariance is present for pictures (e.g., Duñabeitia et al., 2011) and has been found to slow identification processes and to diminish accuracy (e.g., Poldrack et al., 1998; Perea et al., 2011). In addition, we expected that effects of mirror generalization are mainly driven by vertical enantiomorphs (e.g., Kolinsky et al., 2011). In order to exclude that mirror invariance effects in our task may originate from lateral reversals containing more visual overlap than up-down mirror images, the second experiment contained stimuli that were rotated by 90 degrees from its’ axis of orientation.

## Experiment 1

### Methods

#### Participants

Twenty-one university students from Berlin (17 female) took part in the experiment and were monetarily compensated or received partial course credit in exchange for their participation. Their age ranged from 18 to 33 years (*M* = 23.61; *SD* = 3.04). Data of one participant was excluded from data analysis because of high error rates (>20%). The study received ethical approval from the ethics committee of the department of psychology at Universität Koblenz-Landau (former affiliation of RG). They took part based on written informed consent.

#### Materials

Stimuli were obtained from the web-accessible database of Japanese kanji distributed by the University of Oxford, accessible at http://ota.ox.ac.uk/desc/2417. Kanji were selected as to be (a) representative of the kanji in this database in terms of complexity as indicated by the number of strokes (database *M* = 10.34; selected kanji *M* = 10.15), and (b) comparable in complexity (limiting the range of strokes to 7 to 14 strokes). The selected stimuli comprised 400 different single and compound kanji characters (e.g., 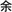 for I/me/my or 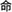 for life/destiny).

**FIGURE 1:**
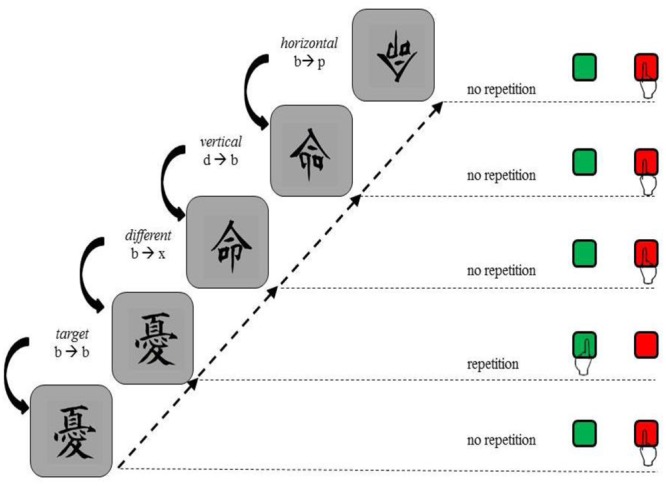
**Procedure and examples of items in Experiments 1.** A *target* relation refers to the repetition of two identical kanji. A d*ifferent* relation refers to the consecutive presentation of two different kanji. A *vertical* relation refers to the consecutive presentation of two identical but vertically mirrored kanji. A *horizontal* relation refers to the consecutive presentation of two identical but horizontally mirrored kanji. Participants should press the green button only if they identified a non-mirrored repetition. In all other cases, participants should press the red button. Kanji as obtained from http://ota.ox.ac.uk/desc/2417.

Four different types of relations between the current and the preceding kanji were possible: (1) *Target*: exact repetition of the same stimulus, for example b/b, (2) *Different:* two different images were presented consecutively, for example b/x, (3) *Vertical*: the second stimulus is a lateral reversal of the prior presented stimulus, for example b/d or p/q, (4) *Horizontal:* the second stimulus is an up-down mirror image of the previous stimulus, for example b/p or d/q. An overview of the design can be found in **Figure 1**.

The kanji were divided into four different sequences and one of the four sequences was assigned to each participant according to participant’s ID. Participants completed seven blocks with 60 kanji (420 trials overall). Each block contained the following repetition types: 10 × *Target*, 40 × *Different*, 5 × *Vertical*, and 5 × *Horizontal*. Using a large stimulus set combined with a low proportion of exact repetitions and mirror-reversed stimuli was meant to minimize the opportunity to practice distinguishing between targets and mirror reversed stimuli over the course of the experiment. The presentation of the kanji was pseudorandomized such that repetitions of the target, as well as repetitions of vertical and horizontal mirror images were excluded. Additionally, each kanji was only assigned to one mirror relation. The kanji characters were presented centrally on the screen in black on a gray background with a character measuring approximately 4° × 5°. The experiment was conducted in MATLAB, MathWorks, Inc., using the Psychophysics Toolbox extensions (Brainard, 1997).

#### Procedure

All participants were tested in individual sessions taking approximately 45 min (including short breaks after each of the six blocks). Participants received a short overview of the session and were seated in front of the computer. After signing informed consent, participants received detailed instructions concerning the assignment of keys (repetition → press green key; no repetition → press red key). The four repetition types were mapped to two different response keys. In case of repetitions with mirror translation or a complete change in stimulus, the red key was to be pressed. Thus, the main challenge in the task was to avoid categorizing the mirror-reversed stimuli as repetitions. Instead, only exact repetitions should be responded with the green key. Participants responded by pressing either the color-covered “d” or “l” key on the second row from the bottom on a standard German PC keyboard. It was accentuated that the <repetition> key should only be pressed if two fully identical kanji were presented consecutively. Participants were also requested to respond as fast and as accurate as possible. The experiment was started by the participant. The kanji were presented for at least 1 s and remained on screen until the participant responded but not longer than 2.5 s. The next kanji was presented immediately after participants’ response or after 2.5 s.

### Data Analysis

Our primary analyses involved two repeated measures analyses of variance (ANOVAs) with repetition type (different vs. target vs. vertical vs. horizontal) as within-subjects factor. In order to correct for violations of sphericity, Greenhouse-Geisser correction for degrees of freedom and *p-*value were used. In order to isolate effects of *vertical* relations, planned comparison with *vertical* repetitions as reference group were conducted and the criterion for significance was adjusted to *p* < 0.01. The $\eta_{p}^{2}$ representing the proportion of explained variance in the dependent variable is reported for each significant effect. The following $\eta_{p}^{2}$ correspond with small (0.10), moderate (0.25), and large (0.40) effect sizes (*f*), respectively: $\eta_{p}^{2}$ = 0.01, $\eta_{p}^{2}$ = 0.06, and $\eta_{p}^{2}$ = 0.14 (Cohen, 1988).

### Results

Because the first trial of each block had no predecessor, it was excluded from analyses. For the analysis of reaction times, all error trials or non-responses (7.8%) were excluded.

**FIGURE 2:**
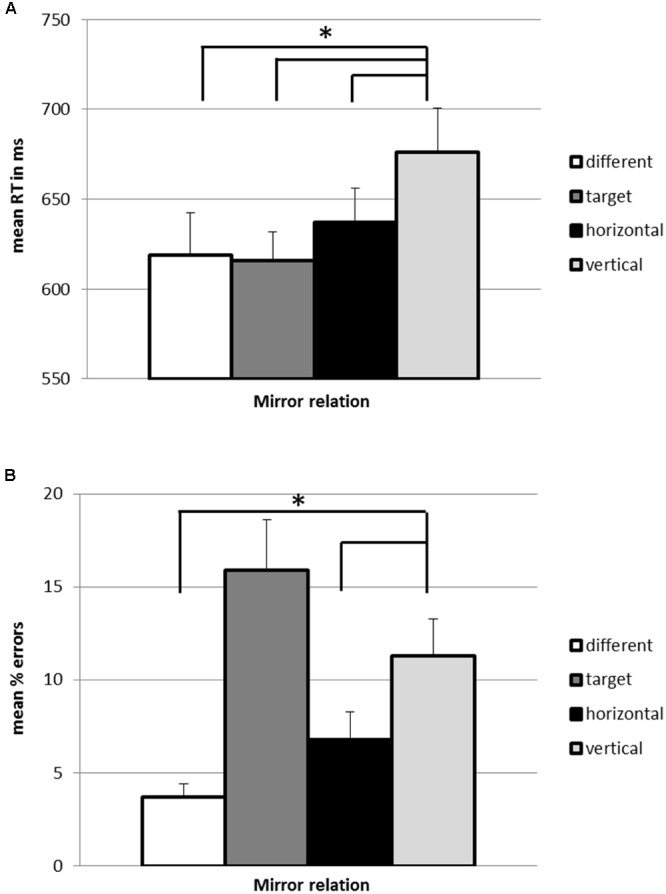
**Mean reaction times (A)** and error rates **(B)** in categorizing stimuli as exact repetitions in the 1-back task in Experiment 1. Error bars depict the standard error of the mean. ^∗^*p* < 0.05.

A repeated measures ANOVA on reaction time revealed a significant effect of repetition type, *F*(3,57) = 7.21, *p* < 0.001, $\eta_{p}^{2}$ = 0.28. Planned comparisons indicated that reaction times for the *vertical* repetitions were significantly slower compared with all other repetitions types. As depicted in **Figure 2**, *different* images (618.66 ms) were responded to faster than *vertical* repetitions (667.12 ms), *F*(1,19) = 18.19, *p* < 0.001, $\eta_{p}^{2}$ = 0.49. *Targets* (615.77 ms) were responded to faster than *vertical* repetitions, *F*(1,19) = 12.99, *p* < 0.01, $\eta_{p}^{2}$ = 0.41. In line with larger reaction time costs originating from more cognitive conflict due to stronger mirror generalization on the vertical as compared to the horizontal axis, *horizontal* repetitions (636.99 ms) were responded to faster than *vertical* repetitions, *F*(1,19) = 8.39, *p* < 0.01, $\eta_{p}^{2}$ = 0.31.

The ANOVA on error rate revealed a significant effect of repetition type as well, *F*(1.72,32.73) = 9.70, *p* < 0.01, $\eta_{p}^{2}$ = 0.34. Planned contrasts showed that *different* images (3.7%) were associated with a significantly smaller error rate than *vertical* repetitions (11.3%), *F*(1,19) = 20.20, *p* < 0.001, $\eta_{p}^{2}$ = 0.52. There was also a significant difference in error rate between *vertical* and *horizontal* repetitions (6.8%), *F*(1,19) = 10.45, *p* < 0.01, $\eta_{p}^{2}$ = 0.36. There was no significant difference between *vertical* repetitions and *targets* (15.9%), *F*(1,19) = 2.17, *p* = 0.16.

**FIGURE 3:**
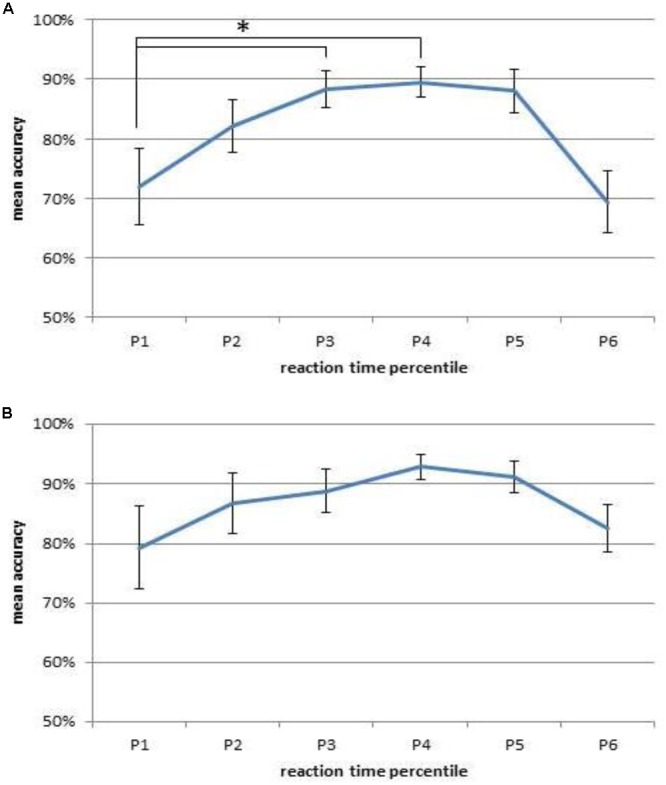
**The conditional accuracy function (CAF) shows the proportion of correct responses to targets for different percentiles of the reaction time distribution (P1: <10% P2: 11–25%; P3: 26–50%; P4: 51–75%; P5: 76–90% P6: >90%) in Experiment 1 (A)** and Experiment 2 **(B)**. Error bars depict the standard error of the mean. ^∗^*p* < 0.05.

Given that targets showed the highest error rate, we analyzed the conditional accuracy function (CAF) for this type of stimulus transition in order to investigate if a speed-accuracy-trade-off occurred. The CAF was computed by ranking each participants reaction times from high to low and partitioning them into six percentiles (*P*1: <10% *P*2: 11–25%; *P*3: 26–50%; *P*4: 51–75%; *P*5: 76–90% *P*6: >90%). In order to test if accuracy was lower for fast responses, a repeated measures ANOVA with percentile as within-subjects factor and accuracy as dependent variable was conducted. The analysis revealed a main effect of percentile, *F*(2.54,48.20) = 6.25, *p* < 0.01, $\eta_{p}^{2}$ = 0.25. To test whether the fastest responses were more error prone than relatively slower responses, planned contrasts with the first percentile as reference group were conducted. As can be seen in **Figure 3** the fastest responses (P1) had a lower accuracy than the third fastest responses (P3), *F*(1,19) = 11.19, *p* < 0.01, $\eta_{p}^{2}$ = 0.37. The fastest responses (P1) also had a lower accuracy than the fourth fastest responses (P4), *F*(1,19) = 9.25, *p* < 0.01, $\eta_{p}^{2}$ = 0.33. None of the other conducted contrasts reached the adjusted level of significance (*p* > 0.01). While, in line with such a trade-off, the fastest reaction times were more error prone than reaction times from the middle of the distribution, the slowest responses showed low accuracy as well.

### Discussion

In Experiment 1 we assessed the effects of mirror generalization for the comparison of the current stimulus to the previously presented stimulus in a 1-back task. We had expected slower reaction times and diminished accuracy in response to lateral mirror reversals. As expected, mean reaction times increased if a mirror inverted stimulus had to be compared to its originally aligned counterpart. As expected, *vertical* repetitions were responded to slower than if two different images were presented consecutively or if an up-down mirror image was presented. Responses to *vertical* repetitions were more error prone than responses to *different* images and response to *horizontal* repetitions.

Contrary to our predictions, responses to *targets* had the lowest accuracy. One possible explanation for this finding is that the number of targets was rather low in each block (1/6th) and, therefore, participants rarely pressed the key. Participants might have habitually pressed the <different> key instead. Only a small number of errors related to the presentation of the *target* were missings (about 12%). However, pressing of the <repetition> key occurred more often for falsely identified repetitions than pressing the <different> key for *targets*. This suggests that categorization failure rather than habitual pressing of the frequently used response key was relevant.

We used kanji stimuli in order to minimize effects of prior experience and to constrain how participants could process the material. For instance, verbal categorization was not likely as participants did not know the verbal labels for the stimuli. Thus, the task allowed to study cognitive conflict caused by memory matching of mirrored visual stimuli. While the kanji helped to avoid that pre-lab practice with the stimuli influenced performance, they were likely not balanced with respect to all visual features potentially relevant for influencing performance. Specifically, it can be suspected that kanji show partial symmetry on the horizontal axis and more so than on the vertical axis. A vertically mirrored kanji might look more similar to the original than a horizontally mirrored kanji would. If, for instance, the amount of lines is not evenly distributed among the upper and lower half of the kanji, a higher level of visual overlap can result for vertically as compared to horizontally mirrored stimuli. This suggests that – alternative to specific effects of vertical mirror generalization – there is another explanation for the stronger effect of vertically as compared to horizontally mirrored kanji in Experiment 1: Vertically mirrored kanji might have stronger visual overlap with the original as compared to horizontally mirrored kanji. In order to address whether stronger mirror invariance effects for vertically mirrored kanji in our task may reflect that lateral reversals contain more visual overlap than up-down mirror images, the second experiment contained stimuli that were rotated by 90 degrees from its’ axis of orientation.

## Experiment 2

Experiment 2 was designed to replicate findings of Experiment 1 and to exclude the alternative explanation of a higher visual overlap as main contributor of the stronger effect of vertical as compared to horizontally mirrored kanji. This was done by rotating the stimuli of three blocks by 90 degrees (clockwise rotation). By this simple manipulation we hoped to reduce visual overlap on the vertical axis. We expected to find a similar pattern of responses in both types of alignments (original vs. 90 degrees).

### Method

#### Participants

Twenty-one university students from Berlin (15 female) took part in the experiment and were paid monetary compensation or received partial course credit in exchange for their participation. Their age ranged from 18 to 35 years (*M* = 26.29; *SD* = 4.26), all but one were right handed.

#### Procedure and Stimuli

Stimuli and procedure were identical to Experiment 1 except that the stimuli in three blocks were rotated by 90 degrees (clockwise).

### Data Analysis

Our primary analyses involved two repeated measures analyses of variance (ANOVAs) with rotation (original vs. 90 degrees) and repetition type (different vs. target vs. vertical vs. horizontal) as within-subjects factors. In order to correct for violations of sphericity, Greenhouse-Geisser correction for degrees of freedom and *p-*value were used. In order to isolate effects of *vertical* relations, planned comparison with *vertical* repetitions as reference group were conducted and the criterion for significance was adjusted to *p* < 0.01.

### Results

Because the first trial of each block had no predecessor, they were excluded from analyses. Data of block one was discarded from data analyses in order to receive an equal number of blocks with two alignment conditions (original vs. 90 degrees). For analysis of the reaction times, all error trials or non-responses (7.8%) were excluded.

**FIGURE 4:**
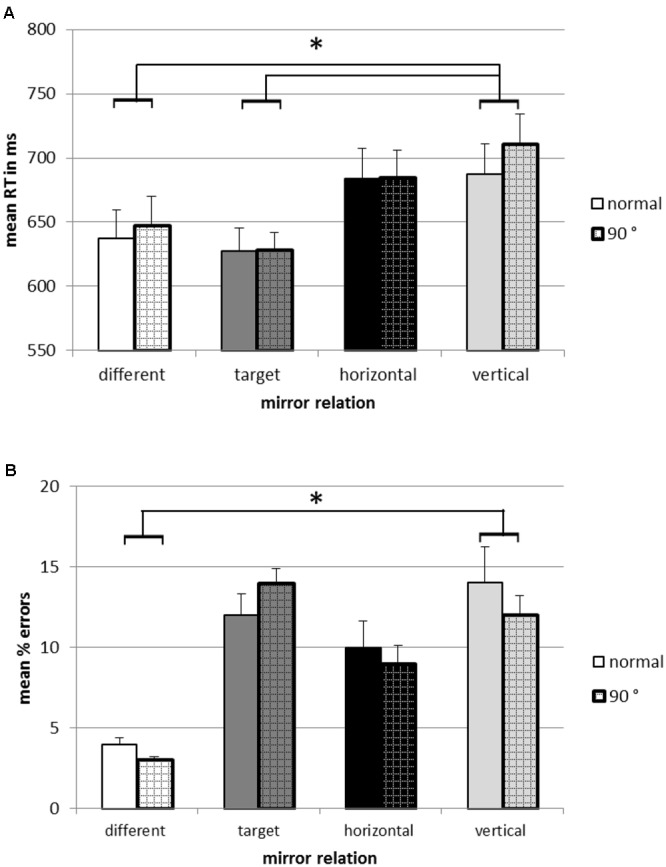
**Mean reaction times (A)** and error rates **(B)** in categorizing stimuli as exact repetitions in the 1-back task in Experiment 2. Error bars depict the standard error of the mean. ^∗^*p* < 0.05.

The repeated measures ANOVA revealed no significant main effect of rotation, *F*(1,20) = 0.89, *p* = 0.36, a significant effect of mirror type, *F*(1.66,33.25) = 8.36, *p* < 0.01, $\eta_{p}^{2}$ = 0.30, and no interaction between rotation and mirror type, *F*(1.93,38.25) = 0.85, *p* = 0.43. As depicted in **Figure 4**, *different* relations (642.19 ms) were responded to faster than *vertical* relations (698.91 ms), *F*(1,20) = 26.68, *p* < 0.001, $\eta_{p}^{2}$ = 0.57. *Targets* (627.66 ms) were responded to faster than *vertical* relations, *F*(1,20) = 10.22, *p* < 0.01, $\eta_{p}^{2}$ = 0.34. However, different from Experiment 1, there was no significant difference in reaction times between *vertical* relations and *horizontal* relations (684.25 ms), *F*(1,20) = 2.09, *p* = 0.16.

The ANOVA on error rate revealed a significant main effect of mirror type, *F*(1.42,28.37) = 3.86, *p* < 0.05, $\eta_{p}^{2}$ = 0.16. There was no significant main effect of rotation, *F*(1,20) = 3.49, *p* = 0.08, and no interaction between rotation and mirror type, *F*(2.21,44.14) = 1.29, *p* = 0.29. Planned contrasts showed that *different* relations (3.1%) were associated with a significantly smaller error proportion than *vertical* relation (10.9%), *F*(1,20) = 16.79, *p* < 0.01, $\eta_{p}^{2}$ = 0.46. There was no significant difference in error proportion between *vertical* and *horizontal* relations (9.2%), *F*(1,20) = 2.31, *p* = 0.10. In addition, there was no significant difference between *vertical* relation and *targets* (12.0%), *F*(1,20) = 0.08, *p* = 0.77.

Again, as target had the highest error rate, a CAF was conducted for this type of stimulus transition. In order to test if accuracy was lower for fast responses, a repeated measures ANOVA with percentile as within-subjects factor and accuracy as dependent variable was conducted. The analysis revealed no main effect of percentile, *F*(2.86,45.70) = 2.61, *p* = 0.08. To exclude the possibility that especially the fastest responses were more error prone than slower responses, planned contrasts with the first percentile as reference group were conducted. As can also be seen in **Figure 3**, none of the contrasts reached the adjusted level of significance (*p* > 0.01).

### Discussion

In this second experiment we tried to replicate findings of Experiment 1, especially the stronger effects for vertical as compared to horizontal mirror images, and to exclude the alternative explanation of visual overlap as main contributor of the observed effects. We expected to find a similar pattern in both types of rotation. Indeed, rotated vs. non-rotated stimuli led to similar reaction times and error proportions. Once again, mean reaction times increased if a mirror reversed stimulus had to be compared to its originally aligned counterpart. However, different from Experiment 1 we did not obtain a specific slowing for vertically mirror reversed as compared to horizontally mirror reversed stimuli. Potentially, including the blocks with the rotated kanji has led participants to use different forms of similarity between presented and memorized stimulus. This suggests that vertical mirror generalization has no exclusive role in automatically influencing the matching between a presented and memorized visual stimulus. Different visual transformations might lead to a level of similarity between memorized and present stimulus that is high enough to cause difficulties in differentiating between stimuli that repeat exactly versus with transformation. Based on the current results we can, on the one hand, state that different forms of mirror generalization are effective in challenging participants’ cognitive control. On the other hand, more work is needed in order to test whether this is only true for mirror transformations or would (for instance) to a similar extent be observed when employing rotations instead of mirror transformations.

In addition, the pattern of differences in error proportion changed slightly. In this experiment, responses to *vertical* repetitions were more error prone than responses only to *different* images. Contrary to the finding of others and ourselves in Experiment 1, there were no differences in error proportions between *vertical* and *horizontal* repetitions (e.g., Kolinsky et al., 2011). Once again, *targets* had the lowest accuracy. This was probably due to differential key press frequency.

## General Discussion

Most visual objects can and should be recognized no matter whether an image is mirror reversed or not. While researchers agree that mirror generalization is a strong and automatic process, they have been disagreeing on whether adults have learned exemptions (i.e., letters like b vs. d, where mirror reversal changes the meaning) to an extent that cognitive conflict should no longer arise (cf. Dehaene et al., 2010). Given the amount of practice adults have accumulated with stimuli not amenable to mirror generalization (i.e., letters), it seems conceivable that they can automatically process such stimuli without engaging cognitive control. Alternatively, adults (usually successfully) suppress mirror generalization with respect to these visual objects if it is not adaptive (e.g., Duñabeitia et al., 2011). Thus, mirror generalization could still lead to cognitive conflict in a way that two mutually exclusive response tendencies are simultaneously present and competing. In fact, a recent study by Borst et al. (2015, p. 228), could show that even “expert readers never completely ‘unlearn’ the mirror-generalization process and still need to inhibit this heuristic to overcome mirror errors.”

In the present study we used visual material that was novel to the participants. While the impact of mirror generalization might be weakened by prior learning with respect to letters (e.g., Kolinsky et al., 2011; Pegado et al., 2014), it should fully impact processing with unknown material such as Japanese kanji. As we wanted to minimize the impact of prior experience, participants were not required to rely on visual long term memory. Rather, our 1-back task involved a comparison of a presented stimulus with one held in working memory, as it had been presented just before (cf. *n*-back task, Jaeggi et al., 2010).

Our results suggest an influence of mirror generalization on performance. Thus, a set-up controlling for prior experience can be used to study how participants can control response tendencies brought about by the automatic process of mirror generalization. Somewhat surprisingly, the results for horizontal as compared to vertical mirroring were inconsistent across experiments. While Experiment 1 was consistent with the special role of vertical mirror generalization suggested in the literature, the stronger effect of vertical as compared to horizontal mirroring was not replicated in Experiment 2 with a procedure addressing a potential confound. Future work, therefore, needs to investigate whether other forms of visual transformations (such as rotation) lead to similar levels of cognitive conflict when comparing a presented and a memorized stimulus.

Consistent with prior studies we have found that in comparison to horizontal reversals, vertical mirror symmetry seems to impede accuracy (e.g., Corballis and Beale, 1976; Bornstein et al., 1981; Wenderoth, 1994; Machilsen et al., 2009; Boldt et al., 2013). However, error rates of vertical vs. horizontal reversals were not entirely consistent across experiments. These contradicting results are in line with the claims of Gregory and McCloskey (2010) who argued that the nature and implications of mirror generalization remain rather unclear as a number of studies could show that horizontal mirror images also have a profound influence on performance (e.g., Huttenlocher, 1967; Sekuler and Houlihan, 1968). In addition, it should be noted that potential impacts of mirroring might more likely rely on object-axis reflection rather than left-right reflections as this effect could be shown in adults and children (Gregory et al., 2011).

Most likely, many different variations of similar pictures can be used to challenge cognitive control. In the current setup, the stimuli which had to be compared were presented sequentially. Potentially, the dynamics of establishing mirrored or rotated representations can account for divergent findings in the literature. For instance, in the current study it is conceivable that due to long presentation times of each stimulus, participants had sufficient time to form horizontally as well as vertically mirrored representations before the next stimulus was shown.

### Limitations and Outlook

As in other studies with the *n*-back task (Jaeggi et al., 2010), response frequencies were not balanced. Most trials contained a stimulus completely different from the one before (2/3rds), requiring the participant to categorize the stimulus as “different.” This response was also due for stimuli that were mirror-images of the n-1 trial rather than exact repetitions (horizontal and vertical mirror variants together 1/6th). These vertically and horizontally mirrored distractors were meant to elicit erroneous “repetition” response tendencies and corresponding delays in RT due to resolving the conflict stemming from automatic mirror generalization suggesting a “repetition” response. The “repetition” response was correct in only 1/6th of the trials (exact repetitions). Using a large stimulus set combined with a low proportion of exact repetitions and of mirror-reversed stimuli, was meant to minimize the opportunity to practice distinguishing between these variants within the experiment.

We cannot quantify effects of unbalanced stimulus- and response frequencies in the current study as we did not vary them. Yet, there are arguments why rare targets and distractors might help to induce cognitive conflict. Filling long waiting times for stimuli that are difficult to discriminate from one-another with distractors has been used to challenge cognitive control in spatial visual attention (Steimke et al., 2016). Furthermore, literature on the proportion congruent effect (e.g., Bugg and Crump, 2012; Dignath et al., 2015) suggests that RT slowing in conflict as compared to no-conflict trials is larger if conflict trials are infrequent. Presumably, participants adopt controlled processing as a default for all trials (conflict and non-conflict trials alike) if conflict trials are frequent. If conflict trials are infrequent, participants seem to rely on automatic processing as default and engage in control processes in the (rare) conflict trials. In addition, work on visual search (Wolfe et al., 2005) suggests that participants seem to miss targets if they are infrequent. At least in part the high rate of misses of infrequent targets seems to be based on problems in inhibiting prepotent response tendencies rather than in problems in visual processing (Fleck and Mitroff, 2007). In that study, participants seemed to press the frequent key often prematurely, despite becoming aware that this was wrong.

While the low target prevalence in our experiments could have led participants to habitually indicate that no target was present, our results rather point to a categorization error (see Discussion of Experiment 1). Note, however, that conditional accuracy analyses suggested that low accuracy was prevalent in trials with very fast and very slow reactions, which at least partially supports the assumption that the low target frequency might have induced a speed- accuracy-trade-off. It would be desirable to test how target prevalence affects target identification processes by varying target prevalence in future work. Moreover, to investigate possible categorization errors due to mirror-reversed stimuli, the present design could be extended by including a third (and probably fourth) key which participants have to press if they think a *vertical or horizontal mirror* relation was presented.

A second limitation is that although mirror generalization is a congenital mechanism and supposed to be existent with comparable strength in (almost) every human being (e.g., Pegado et al., 2011), there may nevertheless be individual differences in this mechanism. More specifically, there may be clear differences between individuals who have received former schooling as compared to individuals who have not. For example, Kolinsky et al. (2011, p. 210) tested unschooled illiterate adults and could show that “learning a written system that incorporates enantiomorphic letters pushes the beginning reader to break the mirror invariance characteristic of the visual system.” Future research should determine to what extent these individual differences influence the extent to which a cognitive conflict arises from mirrored images.

A third limitation is that the current task taxes cognitive control and working memory at the same time. Working memory demands modulate cognitive control (e.g., Jonides et al., 1997; Soutschek et al., 2013). Especially the memory updating procedure influences the conflict adjustment. Future studies should parametrically vary working memory demands in order to follow up on potential interactions of the challenges posed by mirror stimuli and working memory load. As a baseline, load could be reduced to a minimum by employing online comparison of stimuli presented in parallel rather than in sequence. In this setup, a comparison across eye fixations would potentially deliver online process measures of the comparison. We assumed that working memory demands in our study were rather low and given that all our participants were young students, we did not screen participants in terms of additional variables such as working memory capacity or attentional functioning.

In summary, we have shown that mirror transformations of visual stimuli can be employed to challenge cognitive control when comparing stimuli subsequently presented.

## Author Contributions

The work was part of the diploma thesis of MW supervised by RG. MW and RG together developed the research question and research design. MW programmed the experiments, conducted the data acquisition and analyzed the data. MW and RG together wrote the manuscript.

## Conflict of Interest Statement

The authors declare that the research was conducted in the absence of any commercial or financial relationships that could be construed as a potential conflict of interest.
